# Locatable-Body Temperature Monitoring Based on Semi-Active UHF RFID Tags

**DOI:** 10.3390/s140405952

**Published:** 2014-03-26

**Authors:** Guangwei Liu, Luhong Mao, Liying Chen, Sheng Xie

**Affiliations:** 1 School of Electronic and Information Engineering, Tianjin University, Tianjin 300192, China; E-Mails: bhliuguangwei@nankai.edu.cn (G.L.); xie_sheng06@tju.edu.cn (S.X.); 2 Nankai University Binhai College, Tianjin 300270, China; 3 School of Electronics and Information Engineering, Tianjin Polytechnic University, Tianjin 300160, China; E-Mail: chenliying@tjpu.edu.cn

**Keywords:** RFID, temperature sensing, location, remote monitoring

## Abstract

This paper presents the use of radio-frequency identification (RFID) technology for the real-time remote monitoring of body temperature, while an associated program can determine the location of the body carrying the respective sensor. The RFID chip's internal integrated temperature sensor is used for both the human-body temperature detection and as a measurement device, while using radio-frequency communication to broadcast the temperature information. The adopted RFID location technology makes use of reference tags together with a nearest neighbor localization algorithm and a multiple-antenna time-division multiplexing location system. A graphical user interface (GUI) was developed for collecting temperature and location data for the data fusion by using RFID protocols. With a puppy as test object, temperature detection and localization experiments were carried out. The measured results show that the applied method, when using a mercury thermometer for comparison in terms of measuring the temperature of the dog, has a good consistency, with an average temperature error of 0.283 °C. When using the associated program over the area of 12.25 m^2^, the average location error is of 0.461 m, which verifies the feasibility of the sensor-carrier location by using the proposed program.

## 1. Introduction

With the rapid development of wireless communication technology, wireless ways to track and identify a variety of items have become reality [1]. Radio-frequency identification (RFID) technology is highly thought of as an effective tracking and recognition method by more and more people [2]. As the RFID technology makes advances, its application is no longer limited to goods in supply chain management, entrance-guard systems, highway-charge systems [3], *etc.* In recent years, the RFID technology and sensor combination has extended the functionality of RFID systems. Reference [4] reports the use of passive high-frequency (HF) and ultra-high- frequency (UHF) RFID sensors to monitor the humidity and temperature of concrete buildings, to achieve the effect of long-term continuous monitoring that does not need maintenance. In addition, RFID sensors have also been applied in the food cold chain monitoring [5], also for indoor air, temperature and humidity monitoring [6,7]. With the development of economy, and with people's health consciousness constantly improving, the hope that the remote real-time monitoring of human physiological signals for health-related purposes can be implemented in time for the advance disease treatment and intervention, to greatly reduce the medical cost and improve the quality of people's life, is of great significance. Tello, *et al.* [8] proposed a physiological signal remote-monitoring-and-control system, designed to collect information on body temperature, *etc.*, and to communicate with a computer or a mobile device via a Bluetooth module. References [9,10] mention that a wireless body area network (WBAN), specifically centered on Zigbee wireless or Bluetooth technology to access the Internet, can be used to establish contact with a remote medical facility or service companies, for remote monitoring of physiological parameters. The RFID sensors, being small and inexpensive are favored by medical health care [11,12]. Hong Kong's RenAn hospital is successfully using RFID temperature-sensing technology to measure babies' temperature; also Singapore's Tan Tock Seng hospital uses RFID-temperature-sensing technology to measure the patient's temperature. Their system greatly reduces the amount of labor required of medical staff to facilitate the medical treatment of patients.

In recent years, RFID technology with its non-contact, non-line-of-sight, short-time-delay, high-precision and low-cost advantages has been widely used in indoor localization systems [13]. A variety of the RFID technology localization approach [14–19] has appeared which is using the received signal strength indicator (RSSI) methods. Based on its advantages of low power and low-cost, the method has been used in practical location systems, such as LANDMARC [20,21] and the like. This method does not change the reader and the active tag hardware, the only requirement being the design of a special localization algorithm; in what concerns the reader execution algorithm and the related procedures to complete the distance measuring and location [22–24], the method is simple. The main drawback of RSSI methods is poor location accuracy. In order to overcome this shortcoming, the LANDMARC system [21] uses the reference active tag method, whereby the system is mainly composed of multiple readers and a large number of active tags. The active tags in the system have two functions: as location reference points, equivalent to the role of the beacon, called reference active tags, and as positioned active tag. The RSSI method requires a large number of reference active tags to improve location accuracy, but this will increase the cost. In this paper, in comparison with LANDMARC, the system was improved, with a multi-antenna instead of a multi-reader, passive tags instead of active tags, greatly reducing the cost to develop a localization algorithm based on the nearest-neighbor-reference-tag location systems and multi-antenna time-division multiplexing.

Studies on human-physiological-parameter monitoring and on RFID location-technology research have each related reports. However, we have not seen research reports combining the human-physiology-parameter monitoring (such as the body temperature monitoring) and the RFID location. Therefore, this paper presents a real-time system, using RFID technology, for the remote monitoring of body temperature, while at the same time the associated program can determine the location of the body. This approach can be widely used in kindergartens, nursing homes and in the care of other special populations, to achieve early disease detection, and to reduce the incidence. In addition, should major natural disasters (such as earthquakes) and accidents (such as in mines) occur, it could be used for sensing body temperature and for obtaining location information, for timely rescue and thus for reducing mortality and the morbidity which in cases of disaster have an extremely important significance.

## 2. System Design

### 2.1. System Structure and Function

**Figure 1.**
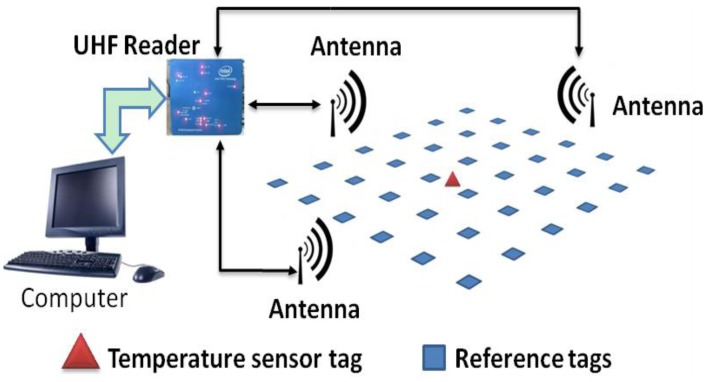
System structure.

This paper presents a use of RFID technology for the real-time remote monitoring of body temperature, while the associated program can also determine the location of the body. A nearest-neighbors localization algorithm is developed based on the use of reference passive tags and of a multi-antenna time-division multiplexing location system. The system structure is shown in Figure 1.

The system hardware includes computers, a UHF reader, RFID-temperature-sensor tags, far-field antennas, and reference passive tags. In Figure 1, placed in accordance with certain rules, are the blue squares representing reference passive tags, and the red triangle representing the RFID-temperature-sensor tag, the tag attached to the human body skin, measuring the surface temperature of the human body. The far-field antennas were placed in three directions outside the region of the reference passive tags, connected by a cable to the UHF reader; they are multiple antennas designed to work via time-division multiplexing. The UHF reader reads the RFID-temperature-sensor tag (conveying the body temperature data) and all the reference passive tags field strength values of the return. The data is transmitted to the computer, the computer runs the associated program using the localization algorithm, and ultimately, through the graphical user interface (GUI), it displays the measured body temperature and location information.

### 2.2. The System Program Structure

**Figure 2.**
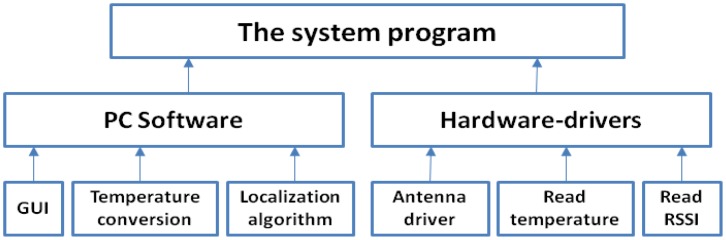
The system program structure.

The system program can be divided into hardware-drivers and PC software; the system program structure is shown in Figure 2. The main function of hardware-drivers program is the implementation of the reader with time-division-multiplexing signals driving three pairs of far-field antennas working alternately. The reader receives the RSSI data from all the reference passive tags and from the RFID temperature sensor tag, stores it in specific registers, then reads the RFID temperature sensor tag data and passes transmitted parameter values to the computer software program. The computer software program includes GUI, temperature conversion, as well as three modules for the localization algorithm; the localization algorithm running on the computer, the location information and temperature data is displayed in the GUI.

## 3. RFID Temperature Sensor Tag Design

### 3.1. The Temperature Sensor Chip

**Figure 3.**
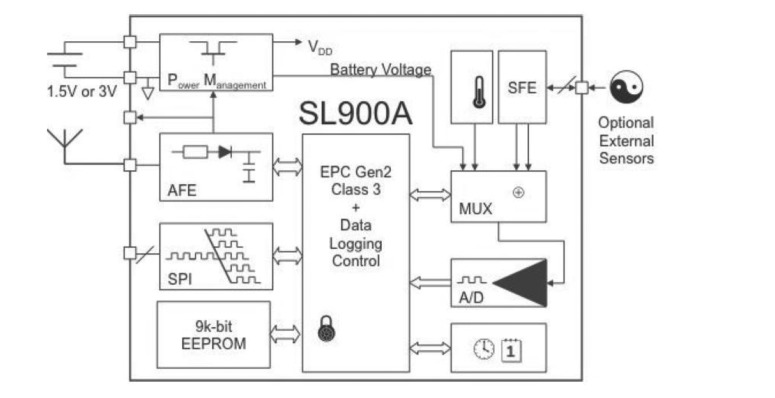
Temperature sensor chip structure diagram.

As in this paper the SL900A [25] chip is used as the core component of the body-temperature monitoring and data transmission part of the experimental system, we show the structure of this temperature-sensor chip in Figure 3. The SL900A single-chip RFID data-logger is a smart semi-active tag with a transponder integrated circuit (IC) that combines a temperature sensor and can automatically both track and record the temperature as well as take readings from an external sensor. The integrated temperature sensor has a typical nonlinearity of ±0.5 °C over the specified temperature range. The device has a real time clock (RTC) and can be configured to notify users automatically in case of an event. It operates in both semi-passive (battery-assisted) or fully passive modes in an ambient temperature range from −40 °C to 125 °C, it transmits at 860–960 MHz, for a battery supply voltage ranging between 1.2 V and 3.6 V, typically of 1.5 V, and has a 9 K-byte EEPROM memory to store the application settings and sensor data, *etc.* By using the RF transceiver circuit in accordance with the communication protocol, the data stored in the radio frequency tag is sent to the reader. The SL900A current consumption in various modes is shown in Table 1.

### 3.2. The Software Settings

**Figure 4.**
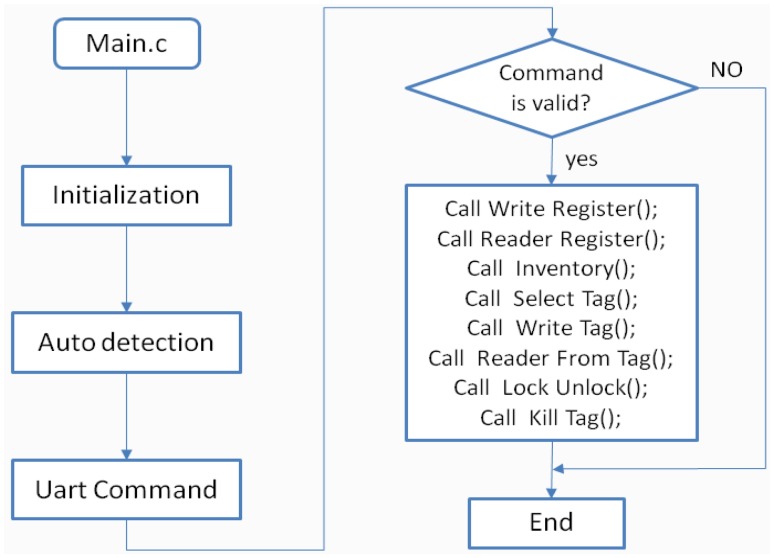
Reader software program flow.

The RFID-temperature-sensor tags operate as both a body-temperature-monitoring device and as a data transfer device. In order to achieve the desired functionality, there is a need for software settings. Parameters can be set by the serial peripheral interface (SPI) or by the RF field (electromagnetic waves from an RFID reader). In this paper, a UHF reader developed by ourselves is used and the parameter setting is done by the RF communication. The reader software program flows as shown in Figure 4. After the reader is initialized, it automatically detects whether a valid serial program is in place. The reader receives a valid command sent from the computer, to perform the appropriate function of the tag to complete the setting. There are very detailed configuration commands in the SL900A datasheet. e.g., the SET LOG MODE command is used to set various parameters, including data storage format, internal temperature sensor enable, external sensors enable as well for the time interval of assigned data collection. The SET CALIBRATION DATA command is used to set the internal-temperature-sensor voltage reference; this paper uses the system default. The temperature-sensor precision is of 0.18 °C. Internal procedures can be initiated by the START LOG command, where the chip will be set accordingly to its detailed programming. Measurement and data recording can also be started by an external trigger, to start the system immediately or with a delay.

## 4. Location System

### 4.1. Location-System Hardware

As mentioned, the work in this paper is based on the reference-tag nearest-neighbors localization algorithm and the multiple-antenna time-division-multiplexing localization system. The hardware part consists mainly of the Intel R1000 RFID development platform, the Impinj far-field antenna, and the Invengo XCTF-8030A passive tag. The Intel R1000 RFID development platform mainly consisting of a microcontroller, integrated transceivers, power amplifiers, RF multiplexer, circulator and coupler and so on, which contains all the internal modules of an RFID reader designed to use RF tag for UHF ClassGen2 inquiry and data reception. This platform can be configured to use both receive antennas (RX) and transmit antennas (TX/RX) antennas and to use both transmit or receive the common mode of operation of the antenna. The Impinj far-field antenna is mainly used for 902∼928 MHz RFID systems, with a center frequency of 915 MHz, in line with the EPC global Gen2 ISO18000-6C standard. It has high sensitivity; the far-field read distance (above 6 m), selectable gain, strong anti-interference and noise reduction capabilities, circular polarization and linear polarization selectable features. The system uses three pairs of Impinj far-field antennas. The R1000 development platform is connected respectively to the TX/RX 0, RX0, TX/RX 1, RX1, TX/RX 3, RX3 ports, where 0, 1, 3 represent the transmitting and receiving antenna of each pair, the TX/RX port is transmitting, while RX is a receive port. Under the control of the platform Intel R1000, three pairs of antennas are used by rotation in a time-division-multiplexing manner.

### 4.2. Localization Algorithm Implementation

In this paper, the localization algorithm based on the nearest-neighbor reference tag has been improved by using, a target-tags historical-trajectory algorithm, as well as a dynamic *k* value setting method and a nearest-neighbor-tags bias-correction algorithm. In the nearest-neighbor localization algorithm, suppose there are *m* reader and *n* reference tags, and *u* target tags. We assume the *n* reference tags are arranged according to a certain distribution on the plane. The *m* readers read the signal strength values for the *n* reference tags and the *u* target tags. These values can be written in the form of a field-strength vector. The field-strength vector of a target tag is denoted *T⃗* = (*T*_1_, *T*_2_, ⋯, *T_m_*), where *T*_j_ represents the field strength value of the respective target tag as perceived by the *j*-th reader; the reference tags field strength value vector is denoted $\vec{R(l)}=\left(R_{1}\left(i\right),R_{2}\left(i\right),\cdots R_{m}\left(i\right)\right)$ where *R*_j_(*i*) represents the read produced by the *j*-th reader to the field strength values of the *i*-th reference tag. To determine the neighboring extent of the reference tags and the target tag, for each target tag, with *p* ∈ [1, *u*], we define:

(1) $$E_{i}=\left|\vec{T}-\vec{R(l)}\right|=\sqrt{\sum_{j=1}^{m}{\left(T_{j}-R_{j}(i)\right)}^{2}}$$

where *E_i_* represents the Euclidian distance between the *i*-th reference tag and the target tag. The smaller *E_i_*, for *i* ∈ [1, n], the closer the two tags. After comparing by size the *E_i_* values, one chooses the *k* nearest distances to the target tag, corresponding to the nearest-neighbor tags, which compose a set κ. This definition of a weight of *k* nearest neighbor tags is introduced:

(2) $$w_{i}=\frac{\frac{1}{E_{i}^{2}}}{\underset{i\in K}{\text{∑}}\frac{1}{E_{i}^{2}}}$$

Thus, by using Equation (3) one can get the coordinates of the target tag:

(3) $$\left(x,y\right)=\sum_{i\in K}w_{i}\left(x_{i},y_{i}\right)$$

Body position changes do not happen a lot in a short time. This justifies the use in this paper of the target tag historical trajectory algorithm, in which the RFID tags worn by the human body as a target tag are associated with historical weighted estimate of the current position. Assuming the current estimated position is (*x*_0_, *y*_0_), and the *i*-th estimated position is (*x_i_*, *y_i_*), where *i* = 1, 2, ⋯, *s*, the estimated value of (*x*, *y*) is obtained after considering the historical track. By investigating the residual weighting function:

(4) $$Q=\sum_{i=1,2\ldots s}\frac{\left|\left[{\left(x-x_{i}\right)}^{2}+{\left(y-y_{i}\right)}^{2}\right]-\left[{\left(x_{0}-x_{i}\right)}^{2}+{\left(y_{0}-y_{i}\right)}^{2}\right]\right|}{{\left(x_{0}-x_{i}\right)}^{2}+{\left(y_{0}-y_{i}\right)}^{2}}$$

when the *Q* value obtained has the minimum value, we can obtain the best estimated value. After derivation, the Equation (4) can be solved for (*x*, *y*):

(5) $$\left(x,y\right)=\sum_{i=1}^{s}w_{i}\left(x_{i},y_{i}\right)$$

where *w_i_* represents the weight of each historical location value:

(6) $$w_{i}=\frac{\frac{1}{{\left(x_{0}-x_{i}\right)}^{2}+{\left(y_{0}-y_{i}\right)}^{2}}}{\overset{s}{\underset{i=1}{\text{∑}}}\frac{1}{{\left(x_{0}-x_{i}\right)}^{2}+{\left(y_{0}-y_{i}\right)}^{2}}}$$

Equation (6) shows, that if the history of the position (*x_i_*, *y_i_*) is closer to the current position (*x*_0_, *y*_0_), then *w_i_* has a larger value, to which it corresponds a larger weight. In actual measurement situations sometimes for several reference tags the field strength values of one or several components of the vector cannot be read, resulting in localization error. The dynamic *k* value method makes more nearest neighbor tags to participate in the RFID temperature sensing tag weighted positioning, in order to reduce the localization error. In the initialization process, the initial number of tag *k*-nearest neighbor is first set to *k*_0_. In the hardware control program, if the value of the field strength component cannot be read, a value is artificially set. In the positioning program, if the number of the detected artificial field strength value tag is *c*, then the number of nearest neighbors tag *k* is corrected to *k*_0_ + *c*. The experiment found that the initial value of *k*_0_ can be 3, 4 or 5, with 4 being better.

The relationship between field strength and distance is not linear, but one similar to a negative exponential curve. When the RFID temperature sensing tag is closer to a reader, steep changes in the value of the field strength, the nearest neighbor tag select deviations and weights deviations, affect positioning results. Therefore, in order to solve the problem we used the nearest neighbor tag positioning error as a correction. Using the method described in the nearest-neighbor localization algorithm, select *k* nearest neighbor tags, and then were consider the *k* reference tags as the target tags, one at a time, the remaining *n* − 1 tags as reference tags, obtained by calculating the estimated position for each target of the tags, denoted ( $x_{i}^{\prime }$, $y_{i}^{\prime }$), where *i* = 1, 2, …, *k*. Then by using the estimated position of the *k* tags and by comparing it to their actual location, get the correction value:

(7) $$\left(\delta x,\delta y\right)=\frac{1}{k}\sum_{i=1,2,\ldots ,k}\left[\left(x_{i},y_{i}\right)-\left(x_{i}^{'},y_{i}^{'}\right)\right]$$

Finally, the correction value is used to obtain the RFID-temperature-sensing-tag location coordinates:

(8) $$\left(x,y\right)=\left(x^{'},y^{'}\right)+\left(\delta x,\delta y\right)$$

**Figure 5.**
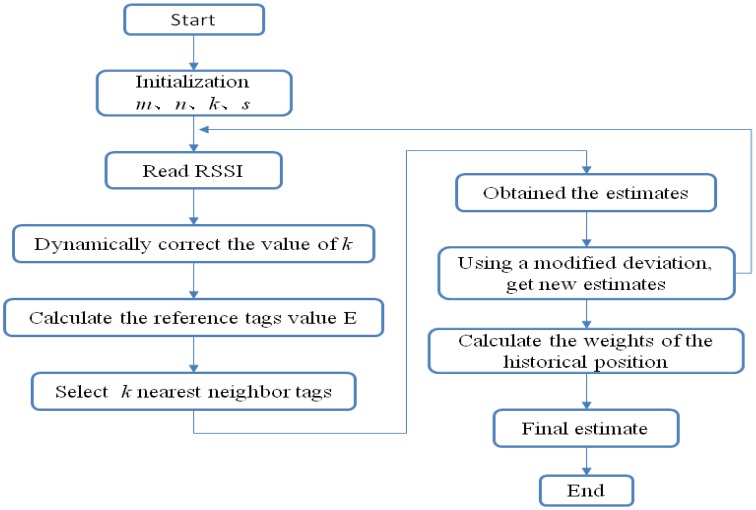
The localization algorithm flow.

The localization algorithm flow is shown in Figure 5.

Following is a brief description of the localization algorithm flow: (1) Set various parameters, such as the number of readers *m*, the number of reference tags *n*, the number of nearest neighbor tags *k*, and historical value reads *s*; (2) Read the value of the field strength of each tag; (3) Correct *k* values in order to compensate for detected failures to read the value of the field strength in the second step; (4) Calculate *E* values for each reference tag; (5) Select *k* nearest neighbors tags according to the value of *E*; (6) Calculate the corrected values for each nearest neighbor tag; (7) Determine the weighted nearest neighbor tag position in order to get an estimate; (8) Repeat (4) to (7), for each nearest neighbor tag as target tag, and the other *n* − 1 as the reference tags, to obtain a location estimate value for each nearest neighbor tag, and the calculated deviation; (9) Get new estimates by making the correction with the deviation of the estimated value of the target tag; (10) Repeat (2) to (9) *s* + 1 times, the last value being the current estimated value, *i.e.*, the historical value before *s* times; (11) Calculate the weights of each historical position; (12) Apply weighting in order to give the final target tag position estimate.

## 5. Results and Discussion

**Figure 6.**
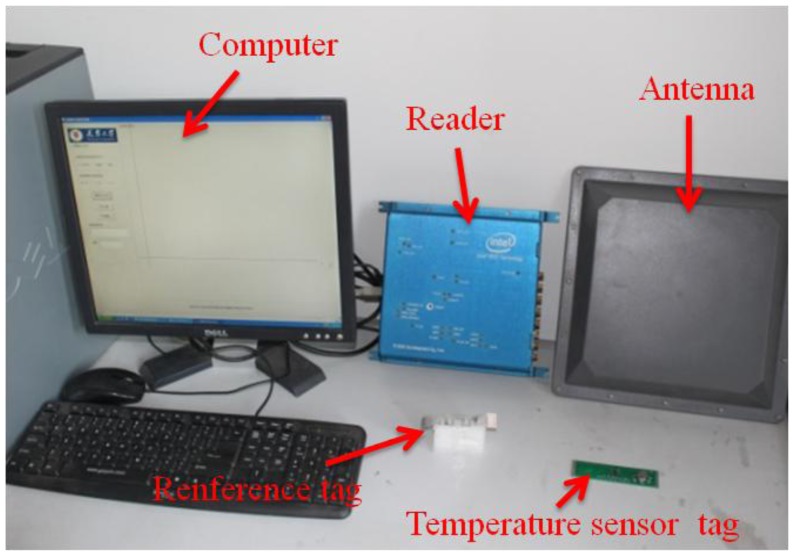
System hardware.

**Figure 7.**
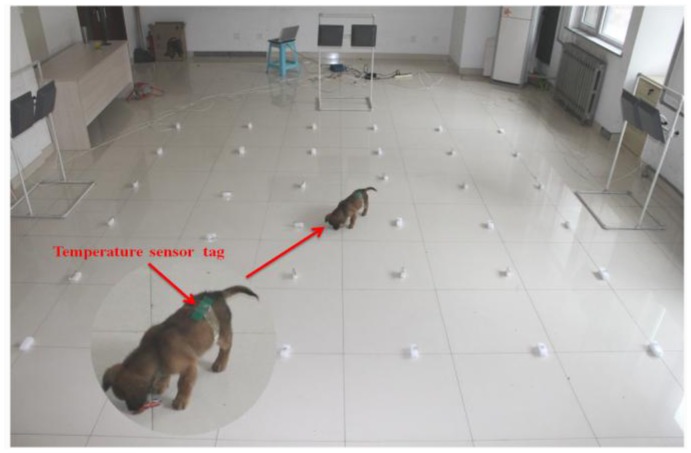
Test site.

In the course of the research described in this paper, an experimental platform for locatable-body temperature sensing was built, based on semi-active UHF RFID tags. As well, an associated program, provided with a GUI, was developed, by using a VC ++6.0 programming environment. The system hardware is shown in Figure 6. The testing experiments were performed indoors, in a room 10.8 m long and 7 m wide. Passive tags were glued on 6-cm-high rectangle foam profiles. Thirty-six of these passive tags were placed in the form of a square array, the distance between passive tags being 70 cm. Three pairs of far-field antenna arrays were placed outside three sides of the square array, which are fixed to the holder at a distance from the ground of 1.2 m, with the tilt angle of 45 degrees toward the square array. Vital signs of a good selection of a dog as a test object for body temperature detection and localization experiments, as well as the test site, are shown in Figure 7.

### 5.1. Temperature Measurement

In the first part of the experiment, a medical mercury thermometer is used to measure the dog's body temperature and its surface temperature in order to obtain their difference. The mercury thermometer is first disinfected with medical alcohol, and then is inserted into the dog's anus for 3 min. After removing the thermometer the recorded value represents the body temperature of the dog. After an interval of 5 min, the above measurements are repeated, for a total of 12 times. The mercury thermometer is again disinfected then placed on the inside of the thigh puppy for 3 min. This gives the value of puppy's surface temperature. After an interval of 5 min, the above measurements are repeated, for a total of 12 times. The measurement results are shown in Table 2. By analyzing the data in the table we see that on average the dog's body temperature is higher by 0.467 degrees than his surface temperature. Next, the dog's body temperature is measured by the RFID temperature sensor tags. A small part of the puppy dog's hair, on its back, is removed to expose the bare skin. The full-contact RFID temperature-sensor tag is applied and fixed with adhesive tape. The dog is placed in the region of the experimental system, with the system software operational. The dog body temperature is measured by wireless, at 3 min intervals, for a total of 12 measurements, the measurement results being shown in Table 2.

**Figure 8.**
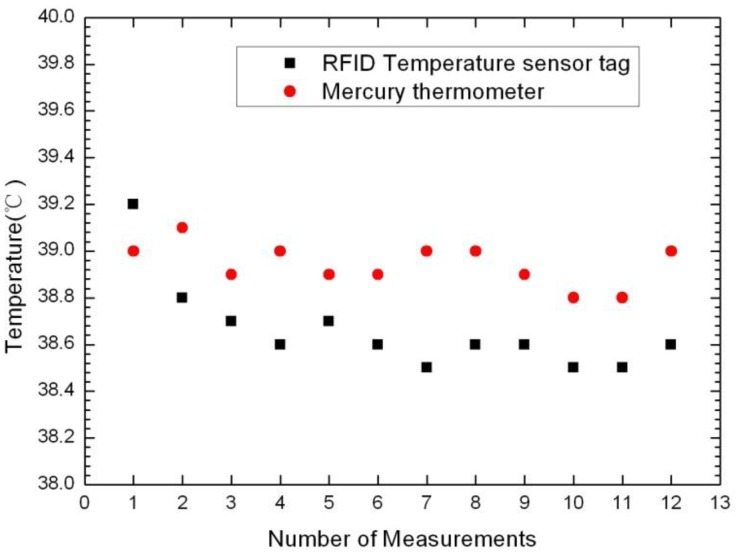
The data analysis results.

Table 2 gives the results of the two different methods for measuring the surface temperature of the dog. The data analysis results are drawn in Figure 8. The black square dots represent RFID sensor-tag temperature measurements, while the red dots represent measurements as given by the mercury thermometer. From the figure it can be found that the position of the red dots is generally higher than the black square dots. This is because when the dog body temperature is measured with a mercury thermometer there is the need to seize the puppies, causing their emotional stress, and leading the temperature to rise. Thus, the results of measurements using the RFID-temperature-sensor tag closer represent puppy's normal body temperature, as the experiment is performed with the dog in a natural state, with more emotional stability.

### 5.2. Localization Accuracy Analysis

**Figure 9.**
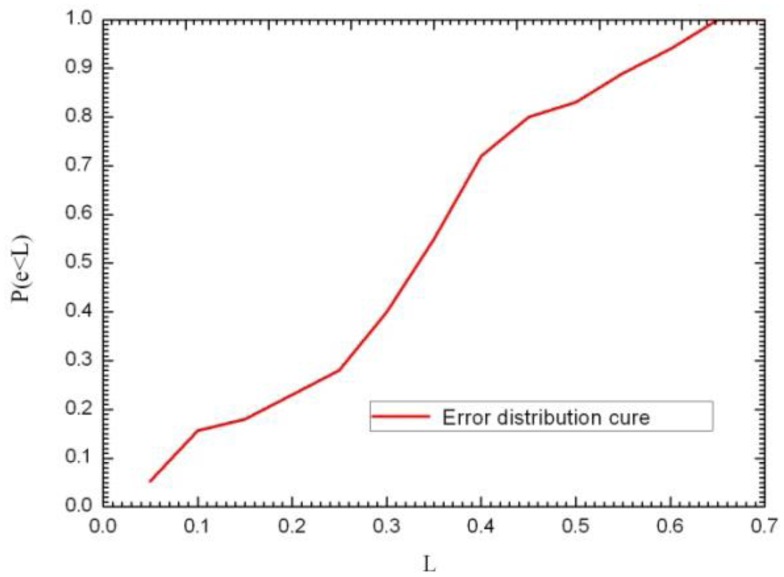
Localization-error probability distribution curve.

In order to study the accuracy of the localization algorithm, the localization error is defined as in formula $e=\sqrt{{\left(x-x_{0}\right)}^{2}+{\left(y-y_{0}\right)}^{2}}$, wherein (*x*_0_, *y*_0_) is the actual coordinate position of the RFID-temperature-sensor tag in the environment, while (*x*, *y*) represents the coordinate position of the RFID-temperature-sensor tag calculated according to the localization algorithm. When using a statistical method for the probability distribution function, *e* is the error in the actual measurement of the position (in meters), and *L* is the abscissa of the probability distribution function (in meters). Subsequently, the measurement error *e* being smaller than a certain level *L* can be shown as a percentage of the total number of measurements in *P* (*e* < *L*). e.g., *P* (*e* < 0.5) shows the percentage of the total number of measurements where the positioning error *e* was less than 0.5 m. The *P* (*e* < *L*) curve was drawn based on the measurement results and is shown in Figure 9.

**Figure 10.**
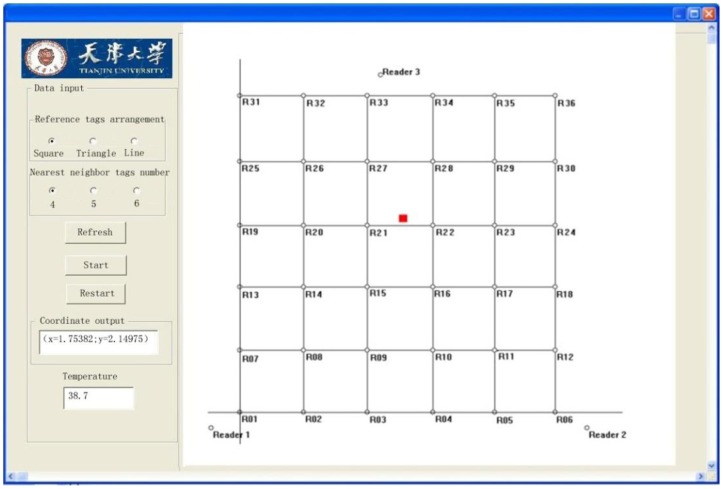
Localization system GUI.

As can be seen from the figure, the probability of a localization error of less than 0.35 m is close to 55%, the probability of an error of 0.4 m 72%, and 83% of the position errors are within 0.5 m. In this study, the average error is 0.461 m; while the maximum error is 0.65 m. Figure 10 shows the localization system GUI. The red dot is the RFID temperature sensor tag's estimated position as given by the localization program. On the left side of the GUI, the spatial-coordinate values as well as the temperature are displayed.

## 6. Conclusions

This paper demonstrates a combined way to perform both RFID real-time remote monitoring of body temperature and location of the respective body. We have not seen research reports combining human-physiology-parameter monitoring (e.g., body temperature monitoring) and RFID location. The RFID chip's internal integrated temperature sensor is used for both the human-body temperature detection and as a measurement device, while using radio-frequency communication to broadcast out the temperature information. The adopted RFID location technology makes use of reference tags together with a nearest-neighbors localization algorithm and of a multiple-antenna time-division multiplexing location system. A graphical user interface (GUI) was developed. Temperature detection and localization experiments were carried out, while using a puppy as test object. The measured results show that the applied method has a good consistency, with an average temperature error of 0.283 °C. Over an area of 12.25 m^2^, the average location error was of 0.461 m. This approach can be widely used in kindergartens, nursing homes and in the care of other special populations, to achieve early disease detection, and to reduce the incidence. In addition, should major natural disasters and accidents occur, it could be used for sensing body temperature and for obtaining location information, which in cases of disaster are extremely important in the timely rescue and thus for reducing mortality and the morbidity.

## Figures and Tables

**Table 1.** SL900A working current in all modes of operation.

**Operating Mode**	**Shutdown**	**Quiescent State**	**RFID Operation**	**Logging Operation**
**Typical current(μA)**	0.1	0.3	50	150

**Table 2.** Measurement results.

**NO.**	**Mercury Thermometer**			**Sensor Tag**

	**Body (°C)**	**Surface (°C)**	**Error (°C)**	**Surface (°C)**
**1**	39.5	39	0.5	39.2
**2**	39.5	39.1	0.4	38.8
**3**	39.4	38.9	0.5	38.7
**4**	39.5	39	0.5	38.6
**5**	39.3	38.9	0.4	38.7
**6**	39.3	38.9	0.4	38.6
**7**	39.4	39	0.4	38.5
**8**	39.4	39	0.4	38.6
**9**	39.5	38.9	0.6	38.6
**10**	39.3	38.8	0.5	38.5
**11**	39.4	38.8	0.6	38.5
**12**	39.4	39	0.4	38.6
